# Correlation of Dopaminergic Denervation and the Progression of Autonomic Dysfunctions in Different Clinical Subtypes of Parkinson's Disease

**DOI:** 10.1155/2021/2268651

**Published:** 2021-11-26

**Authors:** Eun Hye Jeong, Mun Kyung Sunwoo, Sung Wook Hyung, Sun-Ku Han, Jae Yong Lee

**Affiliations:** Bundang Jesaeng Hospital, Department of Neurology, Seongnam, Gyeonggido, Republic of Korea

## Abstract

**Background:**

Autonomic dysfunctions occur in the early stage of Parkinson's disease (PD) and impact the quality of life during the progression of the disease. In this study, we evaluated the serial progression of autonomic dysfunctions between different subtypes of a prospective PD cohort.

**Materials and Methods:**

From the Parkinson's Progression Markers Initiative (PPMI) database, 325 PD patients (age: 61.2 ± 9.7, M : F = 215 : 110) were enrolled. Patients were subgrouped into tremor-dominant (TD), indeterminate, and postural instability and gait disorder (PIGD) subtypes. The progression of autonomic dysfunctions and dopaminergic denervation from I-123 FP-CIT SPECT images of each group were analyzed and compared at baseline, 12 months, 24 months, and 48 months of follow-up periods.

**Results:**

The SCOPA-AUT score of the indeterminate subtype was significantly higher than that of the TD subtype (*P* < 0.05) at baseline and was significantly higher than that of both TD and PIGD subtypes (*P* < 0.05) at 48 months. The indeterminate subtype had the most significant correlation between the aggravation of dopaminergic denervation in I-123 FP-CIT SPECT images and the increase of SCOPA-AUT scores during 48 months of follow-up (*r* = 0.56, *P* < 0.01).

**Conclusions:**

Autonomic dysfunctions were most severe in the indeterminate subtype throughout the 48 months of the follow-up period, with a significant correlation with dopaminergic denervation. We suggest a positive relationship between dopaminergic denervation and autonomic dysfunctions of the indeterminate subtype, beginning from the early stage of PD.

## 1. Introduction

Parkinson's disease (PD) is a neurodegenerative disorder which presents various motor and nonmotor symptoms [1, 2]. Among the heterogenous spectrum of nonmotor symptoms, autonomic dysfunctions affect more than 70% of whole PD patients [3]. It has been suggested that the pathologies of autonomic dysfunctions take place from the very early stage of PD, even 20 years before the appearance of motor symptoms [4]. Moreover, the severity of autonomic dysfunctions even worsens till the later stage of PD [5]. Several previous studies have described the development and risk factors of autonomic dysfunctions during the progression of PD, but are based on cross-sectional designs and have not analyzed the progression patterns nor related pathophysiology [5, 6].

Due to the heterogenetic clinical features of PD, there have been numerous suggestions of categorizing PD into homogenous symptomatic clusters. Analysis of the clinical spectra identified different motor subtypes of PD, such as the tremor-dominant (TD) type and the postural instability and gait disorder (PIGD) type [7]. According to motor-phenotype-based analysis, it has been suggested that autonomic features seem to preferentially affect individuals of the PIGD subtype, especially in the early stage of PD [8–10]. Furthermore, symptomatic cluster-based phenotype analysis of PD revealed that the appearance and risk of autonomic dysfunctions were related with nontremor motor symptoms [11, 12]. Therefore, the underlying pathophysiology and risk factors of autonomic dysfunctions could be accounted on the basis of clinical subtypes, accompanied with analysis of the serial progression of PD.

The aim of this study was to describe the development of autonomic dysfunctions and their correlation with nigrostriatal degeneration during the progression of PD in each subtype. Serial changes of autonomic dysfunctions were evaluated in a prospective cohort. Analysis of the progression of nigrostriatal degeneration was done by periodical quantification of [I-123] N-*ω*-fluoropropyl-2*β*-carbomethoxy-3*β*-(4-iodophenyl) nortropane (I-123 FP-CIT) single-positron emission tomography (SPECT) images.

## 2. Methods

### 2.1. Patients

Data were downloaded in April 2018 from the Parkinson's Progression Markers Initiative database (PPMI, http://www.ppmi-info.org). A total of 325 PD patients (age: 61.2 ± 9.7, male : female = 215 : 110) were analyzed. Patients were enrolled to the PPMI cohort according to the following criterion: patients with PD diagnosis for 2 years or less at the time of screening, patients with proven nigrostriatal dopaminergic deficit on I-123 FP-CIT SPECT imaging at baseline, patients with no history of PD medication within 6 months from baseline, Hoehn and Yahr (H&Y) stage I or II at baseline, and male or females of age 30 or more at baseline. We narrowed down the criteria to patients who had at least two I-123 FP-CIT SPECT image acquisitions and Movement Disorder Society-Unified Parkinson's Disease Rating Scale (MDS-UPDRS) scores and Scale for Outcomes in Parkinson's Disease-Autonomic (SCOPA-AUT) evaluations during follow-up periods at 12, 24, or 48 months from baseline. Patients were subgrouped to the TD, indeterminate, and PIGD subtypes according to the ratio of the mean tremor scores to the mean PIGD scores from the baseline MDS-UPDRS scores (TD patients (ratio ≧ 1.15), PIGD patients (ratio ≦ 0.90), and indeterminate patients (ratios >0.90 and <1.15)) [7]. The PPMI study was approved by each local Institutional Review Board in all participating sites. For all participants, written informed consent for all clinical data was obtained, and written informed consent was given in accordance with the Declaration of Helsinki. All study protocols were performed in accordance with the relevant guidelines and regulations.

### 2.2. I-123 FP-CIT SPECT Imaging

I-123 FP-CIT SPECT images were acquired 4 ± 0.5 hours after I-123 FP-CIT injection (111–185 MBq). Specific binding ratios (SBRs, (target region/reference region)-1) were calculated, with the occipital cortex as the reference tissue, for the right and left caudate and putamen. Among the bilateral SBRs for each caudate and putamen, minimum values were selected for analysis. PMOD software (PMOD Technologies, Zurich, Switzerland) was used for image analysis. Percentage differences (%Diff, 100 × (baseline SBR − follow-up SBR)/baseline SBR) between the baseline and follow-up SBRs were calculated.

### 2.3. Statistical Analysis

Analyses were performed with dedicated software, MedCalc version 19 (MedCalc Software, Belgium). Numeric variables between each group were compared by ANOVA followed by Scheffe's test or by Kruskal–Wallis test followed by Conover test. Shapiro–Wilk test was done to check normal distribution. Frequencies were compared by chi-squared. *P* value <0.05 was considered to be statistically significant. Correlation between quantitative values was evaluated using linear regression analysis and by Pearson's correlation coefficients (*r*).

## 3. Results

### 3.1. Baseline Characteristics

Of the 325 PD patients, 221 patients were subgrouped to the TD subtype (68%), 29 to the indeterminate subtype (9%), and 75 patients to the PIGD subtype (23%). H&Y stage (*P* < 0.001) and MDS-UPDRS Part III score (*P* < 0.05) of the indeterminate subtype were significantly worse, compared with the TD and PIGD subtypes. MDS-UPDRS Part II scores (*P* < 0.001), putaminal SBRs (*P* < 0.05), and caudate SBRs (*P* < 0.01) of the indeterminate and PIGD subtypes were significantly worse, compared with the TD subtype. The SCOPA-AUT scores of the indeterminate subtype were significantly worse than those of the TD subtype (*P* < 0.05), but there were no significant differences between the TD subtype and the PIGD subtype. Baseline clinical features are presented in Table 1. Types of symptoms present at the time of diagnosis were evaluated (Table 2). The PIGD subtype showed significantly lower incidence of resting tremor than other subtypes, and the TD subtype showed significantly lower incidence of bradykinesia than other subtypes.

### 3.2. Aggravation of MDS-UPDRS I Scores, Autonomic Dysfunctions, and Dopaminergic Denervation

The MDS-UPDRS I scores, SCOPA-AUT scores, and caudate and putaminal SBRs were compared between each group at 12 months, 24 months, and 48 months of follow-up periods (Table 3). There were no significant differences of MDS-UPDRS I scores between the groups up to 24 months. After 48 months, the MDS-UPDRS I scores of the indeterminate subtypes were significantly higher than those of the TD subtypes. After 12 months, the SCOPA-AUT scores of the indeterminate and PIGD subtypes were significantly higher than the SCOPA-AUT scores of the TD subtype (*P* < 0.05). After 48 months, the SCOPA-AUT score of the indeterminate subtype was significantly higher than that of both TD and PIGD subtypes (*P* < 0.05). There were no differences of caudate and putaminal SBRs of the three subtypes after 48 months of follow-up.

### 3.3. Correlation of SCOPA-AUT Score Aggravation and Dopaminergic Deterioration during the Follow-Up Period

%Diff of the putaminal SBRs at 48 months of follow-up was correlated with the aggravation of SCOPA-AUT scores (differences of the baseline SCOPA-AUT scores and the SCOPA-AUT scores at 48 months of follow-up). There was a significant positive correlation between the two values in the indeterminate subtype (*r* = 0.56, *P* < 0.01) and the PIGD subtype (*r* = 0.31, *P* < 0.05) (Table 4).

## 4. Discussion

There have been several studies that evaluated the progression and physiologies of clinical symptoms in PD through cluster analysis and categorization of the motor and nonmotor phenotypes [13–15]. According to the study of Erro et al., which is a nonhierarchical cluster analysis also done based on the PPMI database, PD patients were grouped into 3 clusters [15]. Apathy and hallucinations were the most important factors that categorized the patients into 3 groups—the lowest motor and nonmotor group, the motor disability group with apathy and hallucination, and the motor disability group without apathy and hallucination—but no differences of autonomic dysfunctions were found between the clusters. Moreover, the differences of the motor impairment degree between the clusters were relatively mild compared to other cluster analysis studies. In the study of Fereshtehnejad et al., patients were grouped as mainly motor/slow progression, diffuse/malignant, and intermediate clusters [13, 14]. The main motor/slow progression cluster had the highest tremor-related score, while the diffuse/malignant cluster had a significantly higher degree of autonomic dysfunctions. However, these types of cluster analysis are quite difficult to perform in actual clinical practice since it requires complex modeling procedures. In our study, the indeterminate subtype had the worst degree of autonomic dysfunctions at baseline and until 48 months of follow-up. While there were no significant differences of the degree of motor-related symptoms between the three subtypes eventually after 48 months of follow-up, we insist that the indeterminate subtype may be considered as a cluster with the worst autonomic dysfunction-related symptoms. Utilizing the conventional subtyping method would be less troublesome to perform and apply in the clinics, compared with the recently suggested cluster analysis methods.

The aggregation of *α*-synuclein into abnormal Lewy bodies is the well-known hallmark pathology of PD [16]. However, different pathologies are accounted for respective PD symptoms. For instance, the progression of gait disturbance is known to be associated with nigrostriatal dopaminergic denervation due to *α*-synucleinopathy, while the progression of other nonmotor symptoms such as cognitive deterioration is known to be associated with extrastriatal neuronal cell death. Several studies indicated that autonomic dysfunctions are related with *α*-synucleinopathies that start from the autonomic nuclei of the spinal cord and the peripheral autonomic nervous system, extending to the central nervous system [17–22]. This was also revealed by postmortem studies of PD patients with autonomic dysfunctions, which showed that peripheral *α*-synucleinopathy appears in the early stage or prodromal stage starting from the spinal cords [20], which later on affects the substantia nigra and cerebral cortex. The autonomic symptoms are also known to occur in the early stage of PD and may precede the onset of motor symptoms by decades. Therefore, it would be reasonable to consider autonomic dysfunctions as a distinct and major symptomatic cluster of PD other than motor-related symptoms.

The severity of autonomic dysfunctions progresses throughout the advanced stage of PD, with different aggravation rates among subtypes. This can be suggested by the fact that the indeterminate subtype showed a continuous aggravation of the SCOPA-AUT scores until 48 months, while the TD and PIGD subtypes showed almost no changes of SCOPA-AUT scores after 24 months. Also, the SCOPA-AUT scores of the TD subtype showed a relatively higher aggravation rate in-between 12 and 24 months, presumably because of the exponential pattern of symptom aggravation and relatively slower progression of the TD subtype [23]. Furthermore, the MDS-UPDRS I scores of the indeterminate subtype were higher than those of the TD subtype after 48 months. Therefore, our study indicates that the aggravation of autonomic dysfunctions correlates with the deterioration of dopaminergic innervation. This weak positive relationship between putaminal %Diff and SCOPA-AUT score aggravation of the PIGD group in our study may back up several other studies that have revealed the association of autonomic dysfunctions and posture and gait instability phenotypes [11, 12]. Moreover, unlike postural instability and rigidity, tremor does not have a correlation with the accumulation of *α*-synuclein [24] and instead has been suggested to be due to other pathologies such as abnormal neural firing of the basal ganglia [25]. Therefore, we suggest an association with dopaminergic denervation and autonomic dysfunctions, which starts in the early stage of PD, by describing the progression pattern of the indeterminate subtype.

There are some limitations in our study. First, the motor-related symptoms and autonomic dysfunctions were evaluated by MDS-UPDRS scores and SCOPA-AUT scores, respectively, which are questionnaires. Questionnaire score analysis has shortcomings for making accurate diagnosis. Second, the number of the indeterminate subtype was relatively small, and there were differences in age, disease duration, and sample sizes among the subtypes. Third, there could be variations in the I-123 FP-CIT SPECT images since image acquisition was done in multiple institutions. Lastly, our study does not include healthy control groups. Since dopaminergic deterioration is affected by normal aging, serial comparison of the aggravation of SCOPA-AUT and putaminal SBRs of the normal control group may be needed in future studies.

## 5. Conclusions

In conclusion, our study indicates that autonomic dysfunctions may be a major phenotypic cluster, distinct with TD and PIGD subtypes. Moreover, the deterioration of autonomic functions had a strong positive correlation with the aggravation of dopaminergic innervation and starts in the early stage of the disease.

## Figures and Tables

**Table 1:** Baseline patient characteristics.

	TD subtype (*n* = 221)	Indeterminate subtype (*n* = 29)	PIGD subtype (*n* = 75)	*P* value
Age at PD onset (years)	61.2 ± 9.6	62.2 ± 9.1	60.5 ± 10.1	0.78
Gender (male : female)	144 : 78	23 : 6	48 : 17	0.16
Weight (kg)	82.2 ± 17.8	82.8 ± 12.7	79.5 ± 16.6	0.44
Duration of symptoms until study enrolment (months)	61.7 ± 58.4^a)^	73.3 ± 53.8^a)^	46.6 ± 30.8^b)^	<0.05
Hoehn and Yahr staging at baseline	1.5 ± 0.5^a)^	1.9 ± 0.4^b)^	1.6 ± 0.5^a)^	<0.001
MDS-UPDRS Part I score at baseline	2.2 ± 1.6	2.1 ± 1.2	2.7 ± 2.0	0.08
MDS-UPDRS Part II score at baseline	5.9 ± 3.6^a)^	9.4 ± 4.5^b)^	8.0 ± 4.3^b)^	<0.001
MDS-UPDRS Part III score at baseline	20.1 ± 8.6^a)^	25.2 ± 8.9^b)^	20.1 ± 8.0^a)^	<0.05
SCOPA-AUT score	8.4 ± 5.8^a)^	10.6 ± 5.0^b)^	9.4 ± 5.9	<0.05
SBR, putamen	0.7 ± 0.2^a)^	0.6 ± 0.1^b)^	0.6 ± 0.2^b)^	<0.05
SBR, caudate	1.9 ± 0.5^a)^	1.6 ± 0.3^b)^	1.7 ± 0.5^b)^	<0.01
Use of dopamine agonist (%)	56.5	51.7	61	0.63
Average LEDD at 48 months (g)	379.0 ± 245.5	401.9 ± 179.6	359.1 ± 198.5	0.52

For a particular variable, values with different superscripts indicate a statistically significant difference. PD: Parkinson's disease; MDS-UPDRS: Movement Disorder Society-Unified Parkinson's Disease Rating Scale; SCOPA-AUT: Scale for Outcomes in Parkinson's Disease-Autonomic; SBR: Specific binding ratio; LEDD: levodopa equivalent dose.

**Table 2:** Types of symptoms present at diagnosis.

	TD subtype (*n* = 221)	Indeterminate subtype (*n* = 29)	PIGD subtype (*n* = 75)	Total patients	*P* value
Resting tremor	191 (86.4%)	24 (82.8%)	37 (49.3%)	252	<0.01
Rigidity	160 (72.4%)	23 (79.3%)	64 (85.3%)	247	<0.05
Bradykinesia	174 (78.7%)	26 (89.7%)	67 (89.3%)	267	<0.05
Postural instability	12 (5.4%)	7 (24.1%)	5 (6.7%)	24	0.34
Other symptoms	39 (17.6%)	4 (13.8%)	14 (18.7%)	57	0.91

**Table 3:** Aggravation of MDS-UPDRS scores, SCOPA-AUT, and putaminal SBRs during the follow-up of each subtype.

	TD subtype (*n* = 221)	Indeterminate subtype (*n* = 29)	PIGD subtype (*n* = 75)	*P* value
MDS-UPDRS Part I score 12 months	2.5 ± 1.9	2.7 ± 2.3	2.8 ± 1.9	0.39
MDS-UPDRS Part I score 24 months	2.7 ± 2.0	2.9 ± 2.1	3.2 ± 2.7	0.50
MDS-UPDRS Part I score 48 months	3.0 ± 2.4^a^	4.0 ± 2.8^b^	3.8 ± 3.5	<0.05
MDS-UPDRS Part II score 12 months	7.5 ± 4.6^a^	11.0 ± 4.8^b^	9.0 ± 4.6	<0.01
MDS-UPDRS Part II score 24 months	8.1 ± 4.7^a^	11.9 ± 5.3^b^	9.4 ± 5.6	<0.01
MDS-UPDRS Part II score 48 months	10.3 ± 6.0^a^	13.9 ± 6.2^b^	11.5 ± 7.8	<0.05
MDS-UPDRS Part III score 12 months	22.8 ± 10.1	27.8 ± 9.3	23.0 ± 10.7	0.06
MDS-UPDRS Part III score 24 months	24.3 ± 11.2^a^	30.0 ± 11.8^b^	26.6 ± 12.0	<0.05
MDS-UPDRS Part III score 48 months	29.8 ± 11.2	32.5 ± 12.4	30.9 ± 14.2	0.57
SCOPA-AUT 12 months	9.3 ± 7.0^a^	12.6 ± 7.6^b^	11.0 ± 6.7^b^	<0.05
SCOPA-AUT 24 months	12.1 ± 7.0	14.1 ± 6.1	13.1 ± 7.9	0.21
SCOPA-AUT 48 months	12.3 ± 7.3^a^	16.0 ± 7.4^b^	13.4 ± 8.5^a^	<0.05
12-month caudate SBR	1.7 ± 0.5^a^	1.4 ± 0.4^b^	1.5 ± 0.5^b^	<0.01
24-month caudate SBR	1.6 ± 0.5^a^	1.3 ± 0.4^b^	1.4 ± 0.5^b^	<0.01
48-month caudate SBR	1.4 ± 0.5	1.1 ± 0.4	1.2 ± 0.5	0.05
12-month putaminal SBR	0.6 ± 0.2^a^	0.5 ± 0.2	0.5 ± 0.2^b^	<0.05
24-month putaminal SBR	0.6 ± 0.2^a^	0.5 ± 0.2^b^	0.5 ± 0.2	<0.05
48-month putaminal SBR	0.5 ± 0.2	0.4 ± 0.2	0.4 ± 0.2	0.06

For a particular variable, values with different superscripts indicate a statistically significant difference. MDS-UPDRS: Movement Disorder Society-Unified Parkinson's Disease Rating Scale; SCOPA-AUT: Scale for Outcomes in Parkinson's Disease-Autonomic; SBR: specific binding ratio.

**Table 4:** Correlation of putaminal %Diff and SCOPA-AUT score aggravation at 48 months of follow-up.

TD		Indeterminate		PIGD	
Correlation coefficient, *r*	*P* value	Correlation coefficient, *r*	*P* value	Correlation coefficient, *r*	*P* value
0.14	0.07	0.56	<0.01	0.31	<0.05

## Data Availability

The data used to support the findings of this study are included within the article.
